# Catastrophic failure of nacre under pure shear stresses of torsion

**DOI:** 10.1038/s41598-017-13492-z

**Published:** 2017-10-13

**Authors:** Saleh Alghamdi, Ting Tan, Christopher Hale-Sills, Floyd Vilmont, Tian Xia, Jie Yang, Dryver Huston, Mandar Dewoolkar

**Affiliations:** 1 Department of Civil and Environmental Engineering, University of Vermont, Burlington, 05405 USA; 2 Department of Mechanical Engineering, University of Vermont, Burlington, 05405 USA; 3 Department of Electrical Engineering, University of Vermont, Burlington, 05405 USA; 4 Department of Physics, University of Vermont, Burlington, 05405 USA; 5 Department of Civil Engineering, Taif University, Taif, 21974 Saudi Arabia

## Abstract

Nacre, a composite made from biogenic aragonite and proteins, exhibits excellent strength and toughness. Here, we show that nacreous sections can exhibit complete brittle fracture along the tablet interfaces at the proportional limit under pure shear stresses of torsion. We quantitatively separate the initial tablet sliding primarily resisted by nanoscale aragonite pillars from the following sliding resisted by various microscale toughening mechanisms. We postulate that the ductility of nacre can be limited by eliminating tablet interactions during crack propagations. Our findings should help pursuing further insights of layered materials by using torsion.

## Introduction

Nacre is a hierarchical material with extraordinary strength and toughness^1–12^. Composed of ~95% aragonite and ~5% protein, nacreous structures exhibit orders of magnitude higher toughness than their brittle aragonite constituents primarily due to nano- and microscale brick-and-mortar structures^1,4,13–17^. Different toughening mechanisms have been proposed to explain the mechanical behavior of nacre. At the nanoscale, the sliding of nacreous tables are resisted by nanopillars (i.e., mineral bridges)^18–21^, contacts of nanoasperities^20,22–24^ and the unfolding of protein chains^25^. At the microscale, interactions of wavy tablets^26,27^, crack deflections and pulling out of tablets^28^ provide essential resistance to the nacreous deformation. Despite prior studies, it is still difficult to quantify how various nanoscale toughening mechanisms contribute to the shear resistance in the initial sliding of nacreous tablets. It is also unclear how nacre exhibits brittle behavior under different stress conditions. In this work, we employ pure shear stresses of torsion to investigate the mechanical properties of nacre in red abalone, and to further elucidate the evolution of toughening mechanisms during the deformation of nacre.

**Figure 1:**
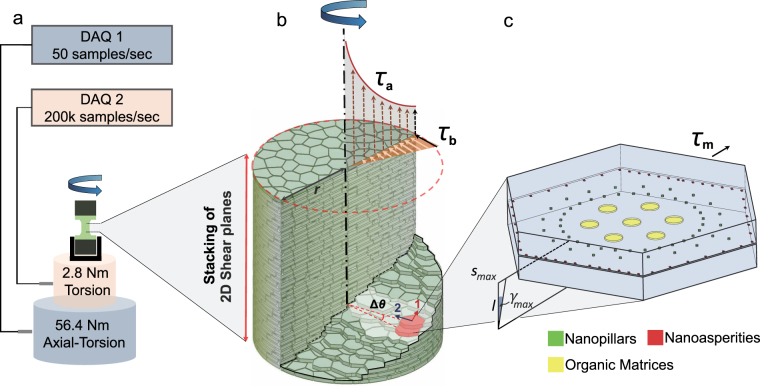
Schematics of the experimental system. (**a**) The dual system that includes an axial-torsional load cell (4.4 kN and 56.4 Nm) at the sampling rate of 50 samples per second and a torsional load cell (2.8 Nm) at the sampling rate of 200,000 samples per second. (**b**) Distributions of pure shear stresses of torsion before (*τ*_b_) and after (*τ*_a_) the failure of nacre specimens. Arrows 1 and 2 denote the sliding and crack directions, respectively. (**c**) A close view of two tablets at the external edge of the gauge section subjected to the maximum shear stress (*τ*_m_) before failure. This geometrical model was used in the finite element analysis.

We focus on separating adjacent nacreous tablets using pure shear stresses of torsion^29,30^. We created composite dog-bone shaped specimens using the pure nacreous sections without any growth layers^21,31–33^ from a red abalone shell. The hexagonal surfaces of aragonite tablets are perpendicular to the cylindrical axis (Fig. 1b). For a shaft under torsion, pure shear stresses exist in every two-dimensional cross-sectional planes over the entire gauge section. This feature enables the nanoscale interfaces between nacreous tablets to be tested successfully. The shear stress and strain in torsion are described by

1 $$\tau =\frac{T\cdot \rho }{J}$$

2 $${s}_{max}={\rm{\Delta }}\theta \cdot r={\gamma }_{max}\cdot l$$

Where *τ* is the shear stress on the horizontal plane of the gauge section, *T* is the torque, *r* is the gauge section radius, *ρ* is the distance between an arbitrary point on the cross section to the center varying from 0 to *r*, *J* = 0.*5πr* ^4^ is the polar moment of inertia, *γ*_*max*_ is the shear strain at the external edge, *s*_*max*_, Δ*θ*, and *l* are the sliding distance at the external edge, twisting angle, and height of two vertically adjacent tablets, respectively. Since nacre specimens are transversely isotropic, shear stresses increase linearly from zero in the center to the maximum in the external edge before the specimen fails. *τ*_*b*_ represents the shear stress distribution before the specimen failure. Once the external aragonite tablets fail, large gradients of shear stresses occur radially due to the rapid decrease in the polar moment of inertia, which is a fourth-order function of the cylindrical radius. These gradients promote fast crack propagations radially until the specimen fails completely. *τ*_*a*_ represents the shear stress distribution after the specimen failure. In this study, the failure of specimens is achieved when the interfacial shear strength is reached. The maximum tablet sliding distance upon failure is measured as the maximum rotational distance between two vertically adjacent brick layers at the external edge. The shear strain is the ratio between the sliding distance and the height of adjacent tablets (Fig. 1c). In the experiments (Fig. 1a), the axial load was precisely controlled (~0.22 N), and the catastrophic failure after peak load was detailed using data collected at a high sampling rate (200,000 samples per second).

## Results and Discussion

### Pure shear stresses generate complete brittle fracture of nacre

**Figure 2:**
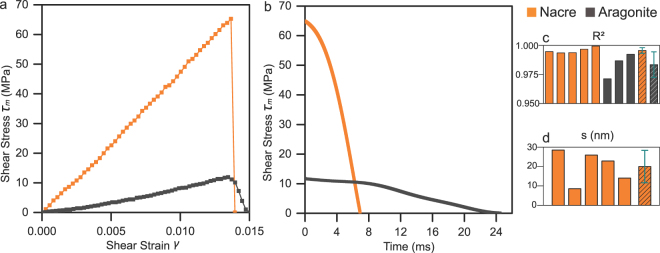
Monotonic torsional tests for nacre and aragonite specimens. (**a**) Shear stress-stress curves. (**b**) Post-peak drop periods with respect to time. (**c**) R^2^ values of the linear-fitting for the increase segments. (**d**) Sliding distances of adjacent nacreous tablets.

**Figure 3:**
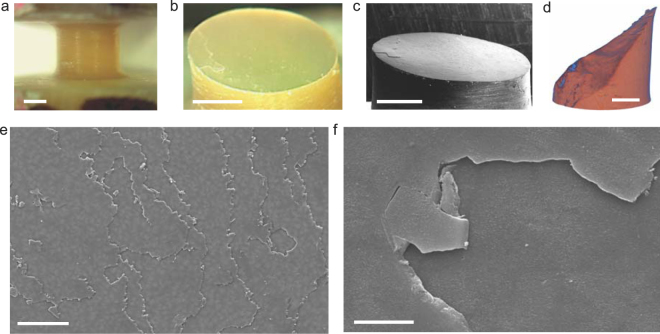
Fractographic characterization. (**a**) A representative dog-bone shaped nacre specimen. (**b**) and (**c**) Optical and SEM images of the fractured nacre half, respectively. (**d**) A microCT image of a fractured aragonite half under torsion, showing a 45° helical fracture. (**e**) A closer view of broken edges and interlayer spiral transitions of nacreous specimens. (**f**) A detail look at inter- and transtablet breakage of nacreous tablets under pure shear stresses of torsion. The scale bars are 1 mm in (**a**), (**b**), (**c**), (**d**), 50 µm in (**e**), and 5 µm in (**f**).

A total of five successful composite dog-bone shaped nacre specimens were created due to the scarce of pure nacre sections in red abalone shells. For each specimen, the torque-rotation curve collected during quasi-static monotonic torsional tests exhibits an increase and a sharp drop (Fig. 2a). The increase segments are almost linear since R^2^ values of the linear fitting are above 0.99 for all specimens (Fig. 2c). The shear strength is 41.5 ± 14.7 MPa at 2.0 ± 0.8% strain. The variation is due to the specimen locations in the red abalone shell. The post-peak curves are nearly vertical, showing that catastrophic failures happen in a short time (Fig. 2a and b). In comparison, single-crystal aragonite minerals were shaped to the same size and orientation of nacreous specimens. The shear strength of aragonite is 14.5 ± 2.2 MPa at 1.4 ± 0.2% strain. Although the R^2^ values of aragonite segments are close to unity, the relatively flat drop (Fig. 2b) indicates that crack propagations are affected by the spiral fractural surfaces (Fig. 3d). For the same geometry, cracks travel faster between the nacreous tablets than in aragonite minerals since the dropping periods of nacre specimens last shorter (5.0 ± 2.0 milliseconds) than those of aragonite specimens (20.0 ± 0.6 milliseconds). By discretizing the twisting angle of the gauge section to the rotational angle of two adjacent tablets, the measured sliding distance at the external edge in the initial stage is ~20 nm (20.0 ± 8.4 nm) that agrees with prior estimations^22^.

### Fractographic characterization proves interfacial fracture in nacre

We observed two flat surfaces over the entire 3 mm-diameter circular cross sections of nacreous specimens after failure (Fig. 3a,c). Under the white light, the flat surface exhibits a combination of green (λ≈510 nm) and yellow (λ≈570 nm) colors, which is different from the iridescence of the inner surface of red abalone shells. The reason is that uniform horizontal tablet (~500-nm thick) layers underneath reinforce lights with specific wavelengths over the entire fracture surface. Elevated tablet sections appear on the left (Fig. 3b and c). This is because pure shear stress condition is no longer maintained once cracks start to propagate after the failure of external tablets. As a result, cracks kink from one interface to another that eventually separate the specimen. Detail SEM image (Fig. 3e) shows that large areas of tablets are exposed and are interlaced with broken edges that transit spirally from one layer to the next^20^. The broken edges (Fig. 3f) include both the smooth intertablet delamination and brutal transtablet breakage. However, these sharp edges are different from the blunted edges of polished nacre specimens^34^, demonstrating that tablets and spiral connections are quickly removed during crack propagations. The microCT image (Fig. 3d) clearly shows the different brittle fracture of aragonite specimens. Aragonite specimens exhibit a classical 45-degree helical fracture perpendicular to the principal tensile stress, while nacreous specimens fracture sharply through the interfaces between aragonite tablets.

### Mathematical modeling quantifies nanoscale toughening mechanisms

**Figure 4:**
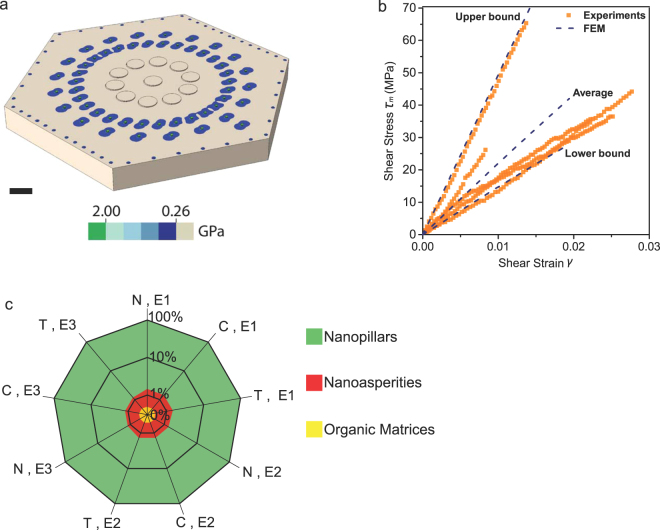
Mathematical modeling. (**a**) Mises stress contours of nanopillars, nanoasperities and organic matrices at the end of the initial sliding. (**b**) Predictions of the upper, lower and average shear stress lines in the torsional tests. (**c**) Contributions of nanopillars, nanoasperities and organic matrices to the shear resistance of nacre in the initial sliding stage. E1, E2 and E3 correspond to the elastic moduli of 100, 90 and 80 GPa, respectively. N, C and T denote zero, compressive (−35 kPa) and tensile (35 kPa) stresses on tablets, respectively. The scale bar is 500 nm in (**a**).

To detail the nanoscale toughening mechanisms in the initial sliding stage, we created isotropic linear elastic finite element models to quantify the contribution of nanopillars, nanoasperities and protein chains to the shear resistance. A contour of an exposed tablet (Fig. 4a) shows that stresses in nanopillars and nanoasperities (E = 100 GPa) are substantially higher than those of protein chains (E = 20 MPa) due to the significantly different elastic moduli. The lower and upper bounds of shear stress curves (Fig. 4b) correspond to nanopillar densities of 1.4 and 5.6/µm^2^, respectively. The mean shear strength (~41.5 MPa) curve corresponds to a density of ~2.2/µm^2^. By decoupling the contribution of each mechanism with respect to various tablet moduli (E = 80 to100 GPa) and pressures (p = −35 to 35 kPa), Fig. 4c shows that nanopillars contribute to more than 95% of the shear resistance, while nanoasperities and protein chains contribute limitedly in the initial sliding stage. High shear stresses in the middle section of nanopillars (~1.0 GPa) enable their breakage at the end of the initial sliding stage. Compared to the shear strength of aragonite mineral specimens (~14.5 MPa), nacreous nanopillars exhibit much higher shear resistance, demonstrating the ‘smaller-is-stronger’ size effect down to the nanoscale.

### The Achilles heel of nacre

**Figure 5:**
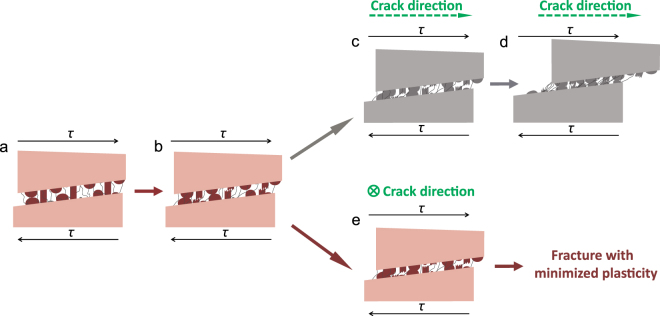
Schematics of the evolution of toughening mechanisms in the nacre deformation. (**a**) The beginning of tablet sliding. (**b**) Initial sliding up to the breakage of nanopillars. (**c**) Decrease in spacing induces more tablet contact. (**d**) Tablet geometries (waviness, pulling out, etc.) contribute to the shear resistance in the microscale toughening stage. Diagram (**e**) shows the direction of the crack propagation is perpendicular to tablet sliding under pure shear stresses of torsion, in which (**c**) and (**d**) are not fully triggered.

If we define the intact nanopillars, nanoasperities and protein chains before sliding as ‘mortar’, the ductile or brittle behavior of nacre is highly dependent on how bricks (tablets) perform after mortar sections fail. The mineral bridges, nanoasperities and protein chains are activated to resist sliding in the beginning. When mineral bridges break after tablets slide about twenty nanometers, vertical distances between tablets decrease (Fig. 5a and b). In most stress conditions, contact areas between tablets increase gradually and substantially as cracks propagate completely or partially parallel to the sliding direction (Fig. 5c). The toughening mechanisms then change to the nanoasperity contact and protein deformation in nanoscale, and to interactions of wavy tablets, tablet pulling-out and crack deflections in microscale^1,27,28^ (Fig. 5d). For example, when nacre is in tension along the tablets^18,24,35^, mortar sections fail first (the linear increment), and some tablets start to touch each other (the nonlinear increment). Then, microscale toughening mechanisms are triggered at various locations continuously (the extended stress plateau). Similar behavior exists in nacre under compression^36,37^, bending^24^, 45-degree shear^24,26^ or direct shear^26,38^. However, when nacre is under torsion normal to aragonite layers, the sliding of tablets (tangential) is perpendicular to the crack propagation (radial), and the large stress gradients enable the specimen to fail quickly (Fig. 1b). Thus, nacre exhibits completely interfacial brittle fracture since there is little chance for brick interactions after the mortar sections fail (Fig. 5e). The Achilles heel of nacre is to avoid triggering microscale toughening mechanisms that can induce the ductile behavior. The discretization nature of torsional loads enable us to study nano- and microscale material behavior using relatively large specimens. Torsion can be particularly advantageous to study interlayer/interfacial mechanical behavior of layered materials.

In summary, by applying pure shear stresses of torsion, we exhibit the linear response in the initial sliding stage of nacreous tablets. Mathematical modeling shows that nanopillars contribute dominantly to the shear resistance, while nanoasperity contact and protein chains contribute limitedly in this stage. Complete brittle fracture observed between tablet interfaces in torsion is convincing proof that microscale toughening mechanisms are not triggered to promote ductile behavior of nacreous structures. These findings open an exciting perspective into studying mechanical properties of natural and artificial layered materials using pure shear stresses of torsion. Future effort could also be extended to study the interactions between nanoparticles that build the nacreous tablets, such as rotations, orders, protein strengthening, and deformation twinning.

## Materials and Methods

### Sample preparation

A red abalone shell with a maximum length of ~210 mm (The Shell shop, CA, US) was obtained to prepare specimens for mechanical tests. The shell was first shaped to 7.6 mm by 7.6 mm cubes, and epoxy ends (Loctite Fixmaster, Rocky Hill, CT) were bonded to increase the gripping area. Using a modified minilathe G8688 (Grizzly Industrial Inc., WA, US) with the high precision control, dry composite dog-bone shaped specimens were created by tuning the cylindrical axis perpendicular to the tablet layers. The gauge sections had diameters of ~3.0 mm and lengths of ~3.0 mm. Then, sample surfaces were smoothed using 400 to 1200 grit sandpapers (3 M Company, Maplewood, Minnesota). A total of five specimens were created using pure nacreous gauge sections that are devoid of growth lines. A similar procedure was followed to create single-crystal aragonite specimens (Gold Nugget Miner online) with diameters of ~3.4 mm and gauge section lengths of ~3.4 mm.

### Torsional tests

Monotonic torsional tests were performed to study the fracture of nacre specimens using an eXpert 8600 Series axial-torsion testing systems (ADMET, Norwood, MA). Displacement control was used to apply the torque at the rate of 90 degrees per minute with a pre-load of ~0.023 Nm, during which quasi-static loading was assumed. Fixtures with four-independent jaws were created to clamp the specimens. A constant tension of ~0.22 N was maintained during the torsional test. The resulting tensile stress was ~35 kPa, which was far below the tensile strength of nacreous tablets^4^. A dual-system was created to detail the torque-rotation curves using two load cells and two data acquisition systems. A small torsional load cell with a maximum torque of 2.82 Nm (Futek, Irvine, CA) was mounted on top of a big axial-torsion load cell (ADMET, Norwood, MA) with a maximum torque of 56.5 Nm and a maximum axial load of 4.45 kN. Signals from the big load cell were collected at 50 samples per second using the MtestQuatro’s system (Admet, Norwood, MA); while signals from the small load cell were collected via a 14-bit U2531A data acquisition unit (Keysight Technologies, Santa Rosa, CA) at 200,000 samples per second. Calibration of the dual system ranged from 0.0025 to 1.24 Nm. The resulting R^2^ value of the linear fitting is 1.0. The twisting angle of the gauge section was measured by subtracting twisting angles of the small load cell and the tapered sections between the clamp end and gauge section from the total rotation recorded. By applying the same series of torque ranging from 0 to 2.25 Nm, twisting angles in the elastic range of an aluminum sample with a 12.7 mm-diameter gauge section were measured using only the big load cell or the two load cells, respectively. The rotation differences were used to establish the torque-rotation relation of the small load cell. Finite element analysis was performed to obtain the torque-rotation relation of the tapered segments. The results show that the twisting angle of the gauge section is ~92% of the total rotation measured.

### Fractographic characterization

Different microscopes were used to characterize fractural surfaces of nacre specimens, including optical, scanning electron and microCT techniques. The optical imaging was conducted using an Aven 26700-300 digital microscope (Aven, Ann Arbor, MI) with the built-in white LED light source. For scanning electron imaging, fractured samples were sputter-coated with gold/palladium film without polishing the surfaces. The coated samples were then examined using JEOL 6060 Scanning Electron Microscope (JEOL USA, Peabody, MA) at the beam voltage of ~20 kV. To compare nacre and aragonite specimens, microCT analysis was performed to scan aragonite samples with the resolution of 6.3 µm at 60 kV and 110 µA for ~6 hours using the Skycan 1173 Xray microtomographer (Microphotonic Inc., Allentown, PA).

### Finite element simulation

Three-dimensional finite element models were created using the ABAQUS software (Simulia, Providence, RI) to simulate the initial sliding between two vertically adjacent tablets on the circumference of the gauge section. Different mechanisms, including mineral bridges, nanoasperities, and organic components, are activated simultaneously in the models. Aragonite tablets were modeled as hexagonal disks with edge lengths of 3 µm and the thickness of 500 nm. The vertical spacing in between was 30 nm. Organic matrices were modeled as cylinders with diameters of ~368 nm, and the total area of matrices occupied 5% of the hexagonal area. Similarly, nanopillars were modeled as cylinders with diameters of 50 nm between tablets. Asperities were modeled as half spheres with diameters of 50 nm and heights of 17.5 nm. Thus, the asperity-asperity inception height was 5 nm, and a normal frictionless contact was assumed for the asperity interaction. When nacre grows, nanopillars extrude between proteins to connect the top and bottom tablets^32,33^. Thus, the contact of nanoasperities was not prevalent before tablets slide. In the models, a maximum of 100 nanoasperities could be placed between two parallel hexagonal edges, 50% of which were assumed to contact their mates on the other tablet. Finite element results show that contact stress between asperities is ~14 GPa when the inception height is 5 nm, i.e., close to the theoretical strength of aragonite (E/10 to E/5, i.e., 10 to 20 GPa)^39^. If the inception height was greater than 5 nm, asperities were assumed to break and were treated as nanopillars. All materials were assumed isotropic, linear and elastic based on the stress-strain curves (Fig. 2a). The models were meshed using 2 to 4 million tetrahedral elements. The elastic modulus for organic matrices was 20 MPa^40^. The elastic moduli for aragonite tablets, nanopillars and nanoasperities were the same, ranging from 80 to 100 GPa^41,42^. The Poisson’s ratios were 0.4 for organic matrices^43^ and 0.3 for aragonite^26^. The bottom tablet was fixed, while the top tablet was constrained except for the sliding and pressure directions. Based on the habitat depths of red abalone^44^, i.e., 3.0–4.5 m and 7.0–10.0 m, residual stresses induced by hydrostatic conditions were assumed ranging from 0, −35 and −70 kPa. When these pressures were superimposed with the tensile stress in experiments (35 kPa), the total stresses applied on tablets were 35, 0 and −35 kPa.
